# An Integrated Framework for Assessing the Value of Community-Based Prevention: A Report of the Institute of Medicine

**DOI:** 10.5888/pcd10.120323

**Published:** 2013-02-14

**Authors:** Nicolaas P. Pronk, Lyla M. Hernandez, Robert S. Lawrence

**Affiliations:** Author Affiliations: Lyla M. Hernandez, Institute of Medicine, Washington, DC; Robert S. Lawrence, Johns Hopkins Bloomberg School of Public Health, Baltimore, Maryland.

Since the early 1900s, the major causes of illness and death in the United States have changed from infectious disease to chronic disease. Recognition is growing that nonclinical community- and population-based prevention has a large role in improving the public’s health and well-being. Health risks such as obesity, tobacco use, and low levels of physical activity are the result of a set of complex, interrelated factors that are difficult to untangle and identify. Health behaviors are important (1), but the importance of such factors as the physical, psychosocial, socioeconomic, and legal environments cannot be overstated (2). Community-based, nonclinical prevention policies and wellness strategies account for as much as 80% of the overall health of a population (3), yet assessing the value of community-based prevention remains challenging and complex. How should the value of community-based prevention be assessed? What should be measured? What should be counted, for whom, over what time period, and how?

To address this issue, the California Endowment, the de Beaumont Foundation, the W.K. Kellogg Foundation, and the Robert Wood Johnson Foundation asked the Institute of Medicine (IOM) to develop a framework for assessing the value of community-based prevention. The charge to the committee included examining the sources of data needed and available for valuing; the concepts of generalization, scaling up, and program sustainability; and the national and state policy implications of implementing such a framework. We provide a brief overview of the report, “An Integrated Framework for Assessing the Value of Community-Based Prevention” developed by the Committee on Valuing Community-Based, Non-Clinical Prevention Programs (4).

## The Concept of Value

Valuing community-based prevention is a complex process. The value of an intervention depends on one’s point of view. For example, public health workers may support a needle-exchange program to reduce HIV transmission rates because evidence indicates its effectiveness (5). However, others in the community may be opposed because they view such a program as facilitating illegal drug use. The trade-offs between benefits and harms are valued differently by each group.

Another important concept in assessing value is the decision of whether or not to monetize the effect of the prevention program. Monetization is not always easily accomplished. Expressing the time resource of a paid worker in dollars is simple, but reflecting the dollar value of increased social cohesion in a community may be impossible.

## The Difficulty in Assessing the Value of Community-Based Prevention

The importance of investing resources to avoid further deterioration of health once a person is ill is generally accepted. It is much more challenging to persuade people to invest resources in programs and services designed to change individual, community, and systems actions before someone becomes sick. Benefits of certain interventions may not apply to the entire population, yet costs are shared and immediate while the benefits are often deferred. Prioritizing among interventions may also be challenging, and disagreements over the relative urgency for 1 program choice over another may hinder decision-making processes.

Community means different things to different people in different contexts and can be defined in many ways. For the purpose of the report, the committee defined it as any group of people who share geographic space, interests, goals, or history. A community offers diverse potential targets for prevention and is often conceived of as an encompassing, proximal, and comprehensive structure that provides opportunities and resources that shape people’s lifestyle (6). A community also provides opportunity for pooling resources that help support initiatives designed to directly or indirectly promote, adopt, and sustain health, regardless whether such initiatives are in the health sector or not. 

## The Domains of Value

The committee identified 3 domains of value (Box). The first domain, *health* (at both the individual and group levels), is an important outcome of interest in community-based prevention. Elements such as education, income, green space, crime, social support, and workplace safety were grouped into the second domain, *community well-being*. Success in community health promotion programs has been related to processes that reflect elements such as leadership, skill building, and civic participation (7). These elements were categorized as *community process*.

**Box. The 3 Value Domains and Examples of Elements and Possible Measures for Assessing the Value for Community-Based Disease Prevention**

**Table:** 

Example of Element	Possible Measure
**Health**	
**Overall**	
Quality of life	Quality-adjusted life year or health-adjusted life expectancy
Perceived health	Self-reported health status
**Physical **	
Mortality (overall and per cause)	Deaths
Morbidity	Rates of conditions or diseases of interest, unhealthy days
Functional capacity	Level of activities of daily living, exercise
Injuries	Rates of injury
**Mental**	
Cognition	Cognitive Abilities Screening Instrument (adult), Dementia Rating Scale (adult), Differential Abilities Scale (children)
Morbidity (depression, anxiety, stress)	Self-reported mentally unhealthy days
Perceived well-being	Self-reported mentally healthy days
Suicide	Rates of suicide
**Community Well-Being**	
**Built environment**	
Land use	Number and quality of facilities — schools, libraries, housing
Transportation	Number of sidewalks for walking, bike paths, buses, metro/trains, automobiles
Building quality (indoor air)	Levels of pollutants (eg, radon, tobacco smoke, chemicals)
Food systems	Grocery stores with healthy choices, farmers’ markets
**Natural physical environment**	
Green space	Parks, preserved open spaces, beauty
**Social and economic environments**	
Social support and social networks	Number, type, frequency of contact
Social cohesion	Trust, respect
Education	Number and quality of schools
Resources	Books, computers, play equipment, class size
Achievement	3rd-grade reading level, high school and college graduation rates
Health literacy	Change in level of health literacy
Employment	Employment/unemployment rate
Safe work places	Physical environment and job effort
Stress	Job demand vs control, job effort vs rewards
Income	Wages, Supplemental Nutrition Assistance Program (SNAP) (food stamps)
Crime and safety	Rates for various crimes
Access to health care and health insurance	Number and type of health care facilities, rate of uninsured
**Community Process**	
Local leadership development	Elected leaders reflect community diversity, number and type of community activists
Skill building	Number and type of peer counselors and community organizers
Civic engagement or participation	Voting rates, volunteering, participation in clubs or other local organizations
Community mobilization	Involvement in civic activities (eg, town hall meetings)

## A Proposed New Framework

The committee concluded that a framework for assessing the value of community-based prevention should meet at least 3 criteria:

1. It should account for benefits and harms in 3 domains: health, community well-being, and community process.
2. It should consider the resources used and compare benefits and harms with those resources.
3. It should be sensitive to differences among communities and take those into account in valuing community-based prevention.

Eight existing frameworks were reviewed to determine their relevance for gathering and processing information to aid intelligent decision making (because a framework for assessing value is embedded within a decision-making context) and, more specifically, whether any of the frameworks met all 3 criteria; none did. Thus, a new framework was created (Figure); it proposes a comprehensive consideration of benefits and harms in the context of health, community well-being, and community process. It also proposes a comprehensive consideration of the resources used to plan, implement, and evaluate prevention interventions.

**Figure:**
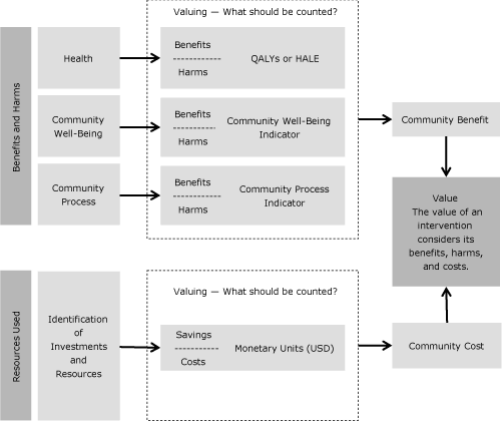
Framework for assessing the value of community-based prevention interventions, 2012. Abbreviations: QALYs, quality-adjusted life years; HALE, health-adjusted life expectancy. Reprinted with permission from the Institute of Medicine.

The goals of the new framework are to 1) incorporate the full scope of benefits into the value of interventions, 2) emphasize that value requires a comparison of the benefits and harms of an intervention with the resources used for that intervention, 3) allow the characteristics and context of each community to be reflected in the valuation of community-based prevention, 4) promote the quantification of value in terms of projected or actual changes effected by the intervention, and 5) encourage the development of evidence to make understanding the effects of interventions easier and more reliable.

## Recommended Action

Measures of health exist (eg, quality-adjusted life years, health-adjusted life expectancy) (8), but measures for the newly introduced domains of community well-being and community process are lacking. Identifying gaps in data sources and developing data sources to fill those gaps are priorities. So is the creation of a metric for community well-being and a metric for community process that could be combined with health measures into a summary indicator of community benefit. Furthermore, decision makers should ensure that elements included in the valuation process reflect the preferences of an inclusive range of stakeholders. A final recommendation reflects the need for transparency to optimize legitimacy of the process.

Value of a proposed community-based prevention intervention is affected by the possible conflict between health inequalities and aggregate health. The degree to which people are willing to trade increased inequality for aggregate improvement may vary significantly, and reasonable disagreement about how to weigh these 2 values may exist. Use of the proposed new framework can make the source for such disagreement more visible. Persistence of such disagreements may suggest a potential legitimacy problem for decision makers. The evidence used for valuation and estimates of the uncertainty of the results should be made public, and decision makers should consider making publicly available the rationales of their decisions.

The new framework is in its early stages, so its near-term effect on policy is likely limited. Expansion of its influence requires building consensus that the proposed domains — health, community well-being, and community process — are all of value in community-based prevention. The data needed to measure tangible benefits adequately are often not available, and the measurement of less tangible benefits is not yet well developed. Good-quality cost data are also important (9,10).

At this time, the committee considers it an important step to promote the use of the framework in the community setting. Although indicators of community well-being and community process are lacking, and so is a summary measure of community benefit, early use of the framework may be useful in identifying all relevant and important elements valued by a community. Those elements should be summarized in outcomes tables and linked to metrics when they are available. Elements that lack metrics should not be left out; rather, a metric should be created, and an attempt at valuing should be made. In addition, we need to validate the framework by showing repeatedly that it correctly distinguishes between interventions that improve value and those that do not. This process will almost certainly require refinement of the framework and expansion of the evidence base.

Although many challenges for comprehensive use of the proposed framework remain, it represents an important step toward realizing the elusive goal of appropriately and comprehensively valuing community-based disease prevention. Use of the framework by communities and decision makers will allow for refinement of the framework and strengthen its value.
